# Evaluation of microbubble contrast agents for dynamic imaging with x-ray phase contrast

**DOI:** 10.1038/srep12509

**Published:** 2015-07-29

**Authors:** T. P. Millard, M. Endrizzi, N. Everdell, L. Rigon, F. Arfelli, R. H. Menk, E. Stride, A. Olivo

**Affiliations:** 1 Department of Medical Physics and Biomedical Engineering, University College London, Malet Place Engineering Building, Malet Place, Gower Street, London WC1E 6BT, UK; 2 Department of Physics, University of Trieste, Via Valerio 2, 34127 Trieste Italy.; 3 Istituto Nazionale di Fisica Nucleare-Sezione di Trieste, Via Valerio 2, 34127 Trieste Italy.; 4 Sincrotrone Trieste SCpA, S. S. 14km 163.5, 34012 Basovizza (TS), Italy.; 5 Institute of Biomedical Engineering, Old Road Campus Research Building, University of Oxford, Oxford OX3 7DQ, UK.

## Abstract

X-rays are commonly used as a means to image the inside of objects opaque to visible light, as their short wavelength allows penetration through matter and the formation of high spatial resolution images. This physical effect has found particular importance in medicine where x-ray based imaging is routinely used as a diagnostic tool. Increasingly, however, imaging modalities that provide functional as well as morphological information are required. In this study the potential to use x-ray phase based imaging as a functional modality through the use of microbubbles that can be targeted to specific biological processes is explored. We show that the concentration of a microbubble suspension can be monitored quantitatively whilst in flow using x-ray phase contrast imaging. This could provide the basis for a dynamic imaging technique that combines the tissue penetration, spatial resolution, and high contrast of x-ray phase based imaging with the functional information offered by targeted imaging modalities.

Advances in medicine are increasingly directed towards targeted interventions and disease specific therapies, with the aim of maximising the benefit to risk ratio for individual patients^1^. Not only does this require detailed understanding of specific disease pathways, to develop effective targeting strategies, but also imaging modalities that enable visualisation of these processes for both diagnosis and treatment monitoring.

Imaging modalities which could be used for this type of functional imaging include nuclear imaging, magnetic resonance imaging (MRI), ultrasound and x-ray based imaging. In each case disease specific information is provided through the use of a contrast agent functionalised to target the relevant tissue process or biomarker^2^. Currently, the nuclear imaging techniques of single-photon emission computed tomography (SPECT), and positron emission tomography (PET), are used routinely for clinical functional imaging.

X-ray imaging offers excellent spatial and temporal resolution, tissue penetration, and is routinely used clinically for diagnostic and, as part of, therapeutic procedures. If functional information could be gained directly from an x-ray based image, then this could be of benefit to a wide range of clinical applications.

X-ray images are conventionally acquired based on the principle of absorption, whereby the x-ray shadow of an object is recorded. A major limitation of conventional x-ray based imaging is that it offers relatively poor soft tissue contrast, particularly compared with MRI. Recent developments based on the use of phase in x-ray imaging have however addressed this. The new techniques instead exploit the phase shift of the x-ray wave front. For clinically relevant materials and energies this is an effect approximately three orders of magnitude larger than absorption^3^, meaning that soft tissue can be imaged at high spatial resolution (<100 μm) with excellent contrast. Phase based methods have also been demonstrated for CT^4^, tomosynthesis^6^ and real time imaging^5^ (fluoroscopy).

Molecular absorption based x-ray imaging has been demonstrated using targeted nanoparticles containing iodine^7^, and more recently gold^8^,^9^. An energy resolving detector can also be used to provide some differentiation between tissue and contrast agent^10^,^11^. An x-ray phase based method would build on this through the greater sensitivity it provides, and the potential for utilising more biocompatible contrast agents.

Microbubbles have been shown to be effective molecular probes for ultrasound imaging^12^,^13^,^14^. They consist of a gas core surrounded by a surfactant or polymer shell, whose surface can be functionalised with a range of targeting species. Microbubbles can also be used as vehicles to deliver therapeutic agents^15^ (either encapsulated or embedded in the shell) enabling both controlled release and enhanced cell uptake of the therapeutic agent through a process termed sonoporation^16^,^17^.

An individual microbubble refracts x-rays acting as an x-ray lens. A population of microbubbles therefore acts to scatter x-rays as a consequence of multiple refraction in many directions. This generates an area contrast when imaged using an XPCi system sensitive to ultra small angle scatter. It has previously been shown that microbubbles are an effective x-ray phase based contrast agent^18^, with contrast enhancement shown with a laboratory x-ray source^19^ as well as with synchrotron based methods^20^.

For effective functional imaging, it is necessary to be able to monitor and ideally quantify, the concentration of a probe within a given region of interest as a function of time. In this study we demonstrate that microbubble concentration can be tracked in real time using x-ray phase contrast imaging (XPCi) under physiologically relevant flow conditions. This development offers the possibility of using XPCi as a modality for dynamic functional imaging of microbubbles, enabling quantification of microbubble concentration in a given volume whilst also providing the required high spatial and temporal resolution, imaging depth and soft tissue contrast. A functional imaging approach based on XPCi has the advantage that a single modality can be used for both high resolution imaging of soft tissue and bone, whilst also providing functional information through the dark field image it makes available^21^,^22^.

## Results

The results presented below demonstrate that a changing microbubble concentration can be tracked quantitatively whilst in flow through a vessel phantom using XPCi. We have previously shown that static microbubble concentrations can be quantified using a ‘single-shot’ XPCi dark field method, whereby microbubbles were imaged in cuboid shaped containers^23^. Images were taken using an analyser crystal based XPCi approach, which is a method that can be extended to other XPCi approaches^24^. A single-shot dark field image refers to an image taken with the majority of the primary beam excluded so that the imaging system is primarily sensitive to x-rays which have undergone ultra-small angle scattering.

A functional XPCi method using microbubbles would be based on imaging the localisation of targeted microbubbles injected into the circulatory system. Monitoring of targeted drug delivery would then be a natural extension of this by embedding a therapeutic agent within the microbubbles.

**Figure 1:**
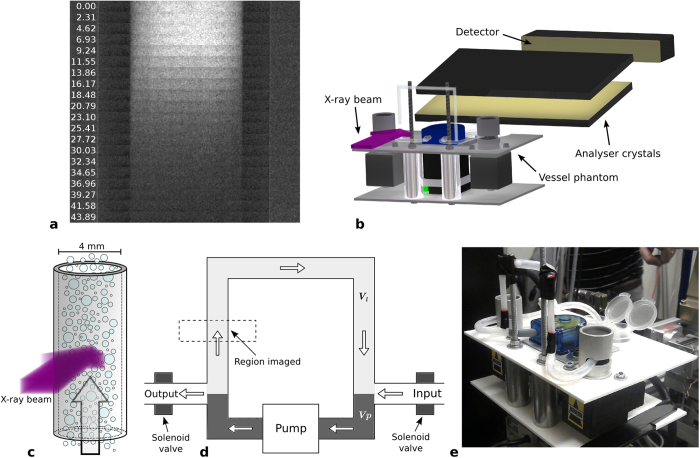
(**a**) Stacked sequence of images taken whilst the microbubble concentration flowing through the tube was reduced. Time (s) elapsed from valve opening is indicated on the left of the figure for each frame in seconds, with all frames displayed using the same grayscale. (**b**) Diagram of the experimental set-up showing the x-ray beam incident on the tubing. (**c**) Diagram depicting how microbubbles were imaged while flowing through the tubing. (**d**) Schematic of the microbubble flow phantom showing the region imaged, the inlet and outlet, and the volumes *V*_*l*_ and *V*p. (**e**) Photograph of the microbubble flow device installed at the SYRMEP beamline at the ELETTRA synchrotron (Italy).

To demonstrate this we show that a varying concentration of microbubbles flowing through a tube (with 4 mm internal diameter to model a vessel, see Fig. 1c) can be extracted from a sequence of x-ray dark field images. Images were continuously acquired as the flowing microbubble concentration was reduced in a controlled manner using a custom designed flow phantom (Fig. 1b–e). For this experimental run the pump flow rate was set to 80 ml/min, and the microbubble concentration decay constant D (see equation 1) was 0.056 s^−1^. A video of this result is shown in supplementary material 1, with Fig. 1a containing a selection of frames from the sequence. The microbubbles make the centre of the tube appear bright in the first frame (taken at 0 seconds). Over time, as the microbubble concentration reduces, the signal intensity produced by the microbubbles also decreases.

**Figure 2:**
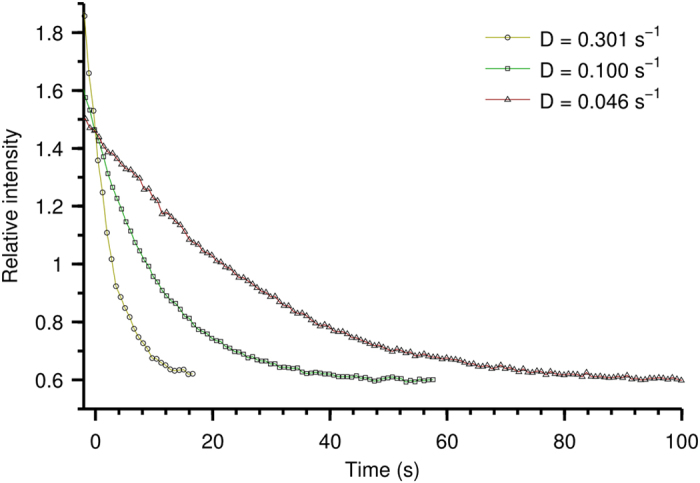
Average intensity calculated in a central region of the tube from three image sequences taken with a different microbubble concentration decay constant (D) for each.

Three image sequences were then collected with a different microbubble concentration decay constant (D) set using a pinch valve, but with the flow rate of liquid through the pump kept constant (250 ml/min). The average intensity in the pixels of the central region of the tube was found for each frame, with the result for each image sequence plotted against time. It proved difficult to achieve precisely the same initial starting concentration for each run, and so zero seconds (*t* = 0) in Fig. 2 is set to a point at which the signal intensity for all three runs overlap. Previous results^23^ demonstrate that this corresponds to identical microbubble concentrations in the tube.

The result of this is shown in Fig. 2, which shows that the rate of decrease in microbubble concentration is related to the rate of decrease in detected intensity. The sequence for which the decrease in microbubble concentration is fastest also shows the fastest decrease in detected intensity. This is expected as the microbubbles generate the signal, and so a faster decrease in concentration leads to faster decay in detected intensity.

It can be seen that, in all three cases, the intensity decays to the same (non-zero) level, due to some residual absorption signal which is also detected when using this single-shot method^23^. When the concentration of microbubbles flowing through the tube becomes close to zero, the intensity detected in all three cases tends to the same asymptotic value.

A Monte Carlo simulation was used to model the signal intensity generated by different concentrations of microbubbles in the tube. The simulation of samples of known microbubble concentration using the previously validated simulation then provides benchmark data^23^.

**Figure 3:**
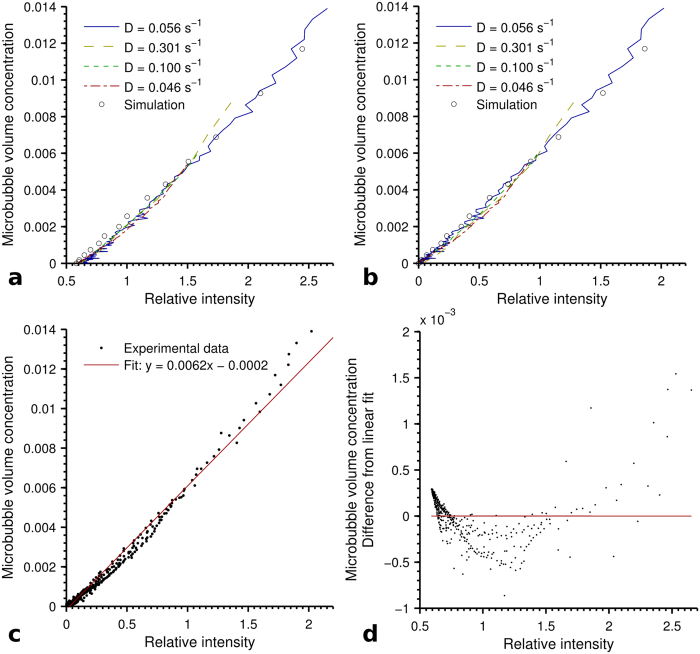
Plots demonstrating how signal intensity is related to microbubble concentration, (**a**) contains data from Fig. 2 where time has been converted to microbubble concentration, and (**b**) the result of subtracting the background intensity. (**c**) Shows the result of a linear fit to the experimental data in (**b**). (**d**) A plot of the residual of the experimental data and fit shown in Fig. 3c, to show the non-linearity of the system response. Markers are the residual, with the line depicting a perfect agreement between experiment and fit.

For each run the microbubble decay constant is known from calibration of the experimental apparatus. Equations 1 and 2 were then used to calculate the microbubble concentration within the tube for each sequence image. This allows a plot of detected intensity against microbubble concentration to be produced, as shown in Fig. 3a. This provides a direct quantitative link between signal intensity and microbubble concentration allowing the latter to be estimated from the former.

Figure 3a contains the simulated benchmark data, as well as the data from Fig. 2 where time has been converted to microbubble volume concentration. It can be seen that all cases show the same trend between intensity and microbubble concentration.

A subtraction method was then used, whereby an image of the tube with a zero concentration of microbubbles (referred to from here as the background intensity) was subtracted from each sequence image, with the results plotted in Fig. 3b. It can be seen that, as a result of this simple correction, zero intensity corresponds to zero microbubble concentration, with the same trend seen as before with increasing concentration.

To give an idea of the sensitivity of such a system to microbubble concentration the signal to noise ratio was calculated for the experimental data shown in Fig. 3b. The Rose criterion states that a signal to noise ratio of five is needed to distinguish a detail^25^. For the experimental data shown in Fig. 3b this corresponds to a microbubble concentration of 0.0017. This value should be treated as a ballpark figure as the system used was synchrotron based, and may not relate to that which could be achieved using a conventional x-ray source. It should also be seen in the context of our simplified phantom structure, and the ‘structural’ noise in a real patient that would make detection more difficult.

It appears as if intensity and microbubble concentration have an approximately linear relationship. Figure 3c contains a plot of the experimental data from Fig. 3b with its corresponding linear fit. A plot of the residuals (Fig. 3d) demonstrates that this is not an exact representation of the trend. However, to a first approximation, this result suggests that for the range of microbubble concentrations used, a linear relation could make the estimation of microbubble concentration more straightforward.

## Discussion

It has been shown that using an XPCi method a changing microbubble concentration can be dynamically tracked in a quantitative manner, which could provide a basis for a functional XPCi technique. As a future development this may allow for XPCi to be used to localise microbubbles targeted towards biological markers, and to measure the concentration of microbubbles at these locations. As well as having use for functional imaging, such an approach could also be used as a means to monitor targeted microbubble therapies. For this technique to be appropriate for clinical applications a number of further technical challenges will need to be addressed.

The experimental results discussed use a single 4 mm internal diameter tube which corresponds, for example, to the diameter of an adult human coronary artery^26^. For use *in-vivo* it can be envisaged that it would be desirable to image vessels of smaller diameter, for which further investigation is required. The sensitivity to microbubble concentration and vessel diameter can be expected to vary greatly depending on the XPCi system used. An investigation of the sensitivity of XPCi signal with varying tube diameter will therefore first require a detailed study of the optimum XPCi system for this application.

A synchrotron based analyser crystal XPCi method was used for the work here, as this method has been well studied and used previously for microbubble imaging^18^,^20^,^23^. An analyser based approach can be used with a conventional x-ray tube source^27^,^28^, but it may not be the most appropriate choice in a clinical scenario. Grating interferometry and edge illumination also both allow dark field imaging, and these three methods need to be studied to find which is best suited^22^,^29^. Potential limitations which need to be studied are sensitivity, exposure time, radiation dose, field of view, system size, and the tolerance of the system to vibration.

Results presented here have been based on planar imaging, and it may be noted that the vessel cross section is needed. This could be solved by using an image processing approach to calculate the cross section of the cylindrical vessels from a projection. It should also be possible to extend the method to a true 3D modality such as a stereographic, tomographic or CT based approach as 3D methods have previously been demonstrated with XPCi^4^.

Further study is also required *ex-vivo* using microbubbles appropriate for clinical use. Here Expancel© (AkzoNobel, Netherlands) microbubbles were used as their polymer shell gave them the stability required for the experiment. Microbubbles were needed which did not burst to allow accurate calculations of microbubble concentration. Signal intensity would be expected to have a dependence on the microbubble size distribution, and so microbubble formulations of different size distributions would be expected to have different relationships with signal intensity. Microbubbles used as an ultrasound contrast agent are required to oscillate to amplify the signal but in XPCi, as this is not needed, microbubbles could be designed with shells of increased rigidity. The microbubble formulation itself could therefore also be optimised to maximise the XPCi signal.

As well as this, investigations are needed to find how targeting agents on a microbubble shell (for example antibodies or magnetic particles^30^) affect the detected intensity. It could be envisaged that the ultra-small angle scatter signal could actually be amplified by nanoparticles embedded in a microbubbles shell, through increased x-ray scatter from these. Nanoparticles have been proposed in combination with microbubbles as a means for targeted drug delivery^31^,^32^ and, though not studied here, since nanoparticles produce an ultra-small angle scatter signal, XPCi could also potentially be used to monitor the concentration of these.

The method proposed has the potential to be used for a diverse range of clinical applications, with the possibilities limited only by the range of biological markers towards which microbubbles can be targeted^33^. The results presented here demonstrate the quantitative measurement of microbubble concentration using XPCi which provides a basis for the development of functional x-ray imaging modalities.

## Methods

### X-ray Phase Contrast Imaging (XPCi)

Samples were imaged using the analyser based imaging (ABI) XPCi method at the SYRMEP beamline of the ELETTRA synchrotron facility (Trieste, Italy)^34^,^35^. An energy of 17 keV was selected by a Si(1 1 1) double-crystal monochromator, with a double crystal analyser arrangement obtained using two single flat Si(1 1 1) crystals. The analyser crystals are placed between sample and detector to convert the phase shifts introduced by the object into a measurable intensity. Images were recorded using a CCD camera with a 9 μm pixel size (Photonic Science, Robertsbridge, UK). The crystal rocking curve position was measured as the fraction of the readings of ionisation chambers placed either side of the analyser crystals to give the crystal reflectivity.

All image sequences were acquired using the single-shot dark field method^23^, with the analyser crystal positioned at the tail of the crystal rocking curve so that 5% crystal reflectivity is seen. Images were acquired with a 0.5 second exposure time, with 4 × 4 detector binning used to decrease read out time. In this mode the detector had a read out time of 0.27 seconds, which gave a frame rate of 0.77 seconds. In all cases intensity was averaged from a region in the centre of the tube.

### Sample Preparation

For this proof of principle work microbubbles were required which are stable for a sufficiently long period. Polymer shell Expancel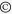 (AkzoNobel) microbubbles were used, which have a copolymer shell of acrylonitrile and vinylidene chloride and a median radius of 4 μ*m*.

Flowing microbubbles were imaged in ultrasound coupling gel (Anagel, Ana Wiz Ltd., Surrey, UK), which predominately consists of water with propylene glycol and glycerol. The gel was diluted to lower the viscosity and allow for pumping and mixing of the flowing liquid. This was prepared by mixing one part ultrasound coupling gel to four parts water, with this ratio chosen as it was found experimentally to be viscous enough to allow for a homogeneous distribution of microbubbles in the flow device, whilst also allowing for mixing during the flow devices dilution action. *In-vivo* microbubbles would be imaged flowing in the circulatory system, where they will be held in suspension in blood. It should be noted that blood is more viscous than water, and this gel solution was designed with this in mind.

An experiment was performed where the gel was imaged in water using the same experimental set-up, which showed that the gel was indistinguishable from water.

### Flow Device

The experimental apparatus used was custom designed for this experiment as a means of flowing microbubbles at physiologically relevant flow rates through the imaged section of tubing. The device (shown in Fig. 1e) also made it possible to dynamically decrease the concentration of the flowing microbubbles.

The device consists of a series 200 mini peristaltic pump fitted with 5 mm internal diameter silicone tubing and powered by a stepper motor (Williamson Pumps Ltd, Poynings, UK) which allowed for accurate control of flow rates up to 250 ml/min. A loop of tubing was then created between the inlet and outlet of the pump, with a part of this being the 4 mm internal diameter Nylon-12 tubing through which the microbubble containing liquid was imaged. Nylon-12 was chosen as it produces a minimal ultra-small angle scatter signal and similar x-ray attenuation to water.

At the two junctions between pump inlet/outlet and the loop tubing a further two pieces of tubing were connected, which function as an input and output to the loop. A solenoid pinch valve (Sirai, Bussero, Italy) was located on each of these, close to the loop, which allows for the input/output of the loop to be opened/closed. The loop is filled with a high microbubble concentration liquid, which is then pumped around the loop at the desired flow rate with the valves closed. A container of liquid without microbubbles is placed on the input tubing, and the valves opened. The differential pressure between the pump inlet and outlet results in this microbubble free liquid being drawn into the loop, thus decreasing the flowing microbubble concentration. This rate of dilution is continuous, and can be accurately controlled by pump flow rate and changing the diameter of the input tubing using a pinch valve. A schematic of this system is shown in Fig. 1d.

The system was controlled via a data acquisition (DAQ) device (NI USB-6008) and LabVIEW software (National Instruments, Texas, US).

When the valves are opened the microbubble concentration flowing through the loop decreases at an approximately exponential rate. This assumption can be made as the length of the loop tubing is short relative to the combination of pump flow rate and detector exposure time. If the initial concentration flowing around the loop is *C*_1_, and the concentration after time *t* is *C*t then


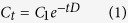


where,


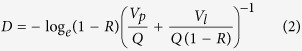


Here Q is the pump flow rate (ml/s), *V*_p_ is the volume of loop tubing on the pump side of the loop junction (2.3 ml) and V_*l*_ on the opposite side of the loop junction (2.8 ml). *R* is the flow rate through the output tube divided by the flow rate through the pump.

### Simulation

The computational model used to simulate microbubble XPCi was a Monte Carlo model developed using McXtrace^36^, which we previously validated for microbubbles^23^.

Using the experimentally measured size distribution, 3D numerical microbubble phantoms were created with a range of concentrations. These numerical phantoms consisted of a random 3D arrangement of polydisperse spheres within a hollow cylinder with wall thickness to match the Nylon-12 tubing. The real and imaginary components of the refractive index for each material were taken from xraylib^37^, with materials Nylon-12 and water used to model the tube wall and liquid within the cylinder respectively.

The simulation was performed as follows. A parallel plane wave of 17 keV photons was incident on the numerical phantom. These pass through the sample, being attenuated and deflected at boundaries according to the principles of ray optics. Directly after the sample the direction of the wave is finely sampled, with this then convolved with the experimentally measured analyser crystal rocking curve. The signal at a crystal rocking curve position can then be found on a pixel by pixel basis from this result.

## Additional Information

**How to cite this article**: Millard, T. P. *et al.* Evaluation of microbubble contrast agents for dynamic imaging with x-ray phase contrast. *Sci. Rep.* **5**, 12509; doi: 10.1038/srep12509 (2015).

## Supplementary Material

Supplementary material 1
